# Real-world clinical results of CGRP monoclonal antibody treatment for medication overuse headache of migraine without abrupt drug discontinuation and no hospitalization

**DOI:** 10.1016/j.heliyon.2024.e40190

**Published:** 2024-11-06

**Authors:** Takafumi Tanei, Yutaro Fuse, Satoshi Maesawa, Yusuke Nishimura, Tomotaka Ishizaki, Yoshitaka Nagashima, Manabu Mutoh, Yoshiki Ito, Miki Hashida, Takahiro Suzuki, Syun Yamamoto, Toshihiko Wakabayashi, Ryuta Saito

**Affiliations:** a Department of Neurosurgery, Nagoya University Graduate School of Medicine, Nagoya, Aichi, Japan; b Department of Specialized Headache Outpatient, Nagoya Garden Clinic, Nagoya, Aichi, Japan; c Academia-Industry Collaboration Platform for Cultivating Medical AI Leaders (AI-MAILs), Nagoya University Graduate School of Medicine, Nagoya, Aichi, Japan; d Department of Neurosurgery, Nagoya Medical Center, Nagoya, Aichi, Japan

**Keywords:** Migraine, MOH, CGRP, Monoclonal antibody, Discontinuation, Hospitalization, Acute medication use

## Abstract

**Background:**

Abrupt discontinuation of overused medications is standard treatment for medication overuse headache (MOH), but discontinuation is difficult to maintain. The aim was to evaluate the real-world clinical results of anti-calcitonin gene-related peptide monoclonal antibody (CGRP-mAb) treatment for migraine with MOH without abrupt drug discontinuation and no hospitalization.

**Methods:**

Data were collected before starting CGRP-mAb injections (baseline) and 1 month after each injection. The following items were compared between baseline and after the first, second, and third CGRP-mAb injections, monthly headache days (MHD), monthly migraine days (MMD), monthly acute medication use (AMU) days, monthly total amount of AMU tablets, headache impact test-6 (HIT-6), and the migraine-specific quality of life questionnaire (MSQ). Achieving reduction rates ≥50 % in the frequency of each headache and migraine was defined as a good response. Achieving reduction rates of both AMU days and tablets ≥50 % was defined as effective in reducing AMU.

**Results:**

This study included 33 patients with migraine with MOH. After the third CGRP-mAb injection, MHD and MMD were significantly decreased from median 30.0 to 9.5 days, and 10.0 to 1.5 days, respectively. In addition, monthly AMU days and tablets were significantly decreased from median 28.0 to 8.0 days, and 30.0 to 9.5 tablets, respectively. After the third CGRP-mAb injection, the good MHD and MMD responder rates were 75.0 % and 85.7 %, respectively. The rate of reducing AMU was 78.6 %. HIT-6 and MSQ scores decreased significantly from baseline to after each CGRP-mAb injection.

**Conclusions:**

When CGRP-mAb was administered to migraine with MOH, frequency of headache symptoms and AMU were reduced without abrupt drug discontinuation and no hospitalization.

## Highlights

• Real-world clinical results of CGRP-mAb treatment for MOH of migraine without abrupt drug discontinuation and no hospitalization.
• Comparing baseline and after three CGRP-mAb injections, monthly headache days and acute medication use were significantly decreased.
• CGRP-mAb treatment may allow patients to withdraw from MOH without abrupt drug discontinuation or hospitalization.

## 1 Introduction

Migraine is a common disabling neurological disorder and it is estimated that more than one billion people worldwide suffer from migraine headache [1]. Migraine headaches are often accompanied by severe headache symptoms, vomiting, and hypersensitivity to various stimuli that can limit participation in daily activities. Migraine is classified as episodic or chronic; chronic migraine can progress to medication overuse headache (MOH), which is caused by chronic overuse of acute medications for the treatment of headache symptoms [2,3]. The annual prevalence of headache owing to MOH is 1–2%, and its prevalence in the general population is reaching 1–7% worldwide [2,[4], [5], [6]]. Combination analgesics and triptans are the drugs commonly associated with MOH, and migraine is a more common primary headache cause of MOH than tension-type headache [7]. Risk factors for MOH include as female gender, psychiatric comorbidities, pre-exisiting pain and medication use, and lifestyle-related factors, with chronic migraine being particularly vulnerable to MOH [7]. The management of MOH consists of abrupt discontinuation of overused medications and initiation of preventive treatment [2,[8], [9], [10], [11], [12]]. This should be the best approach and has long been the chosen treatment strategy [2,11,13,14]. A randomized clinical trial comparing of 3 treatment strategies for MOH reported that withdrawal therapy combined with oral prophylactic medication from the start of withdrawal is recommended as treatment for MOH [14]. However, there are two issues with this treatment method. The first issue is that abrupt drug discontinuation may cause escalating headache and withdrawal symptoms. In such cases, supportive therapy using levosulpiride, metoclopramide, paracetamol, steroids, or benzodiazepines may be required [15,16]. The second issue is that it is realistically difficult for patients with MOH to stop taking overused medications on their own without providing an environment where acute medications cannot be used. Therefore, to complete this treatment method, patients may need to be hospitalized. However, many patients with MOH are middle-aged, and they are often involved in work or childcare [4,5,17]. They are less likely to accept hospitalization for migraine treatment. Furthermore, in Japan, due to a lack of social understanding of migraine, it is difficult to gain social consent and support for hospitalization for the purpose of migraine treatment.

Patients with chronic migraine or migraine with MOH may already be taking conventional oral prophylactic medicines such as anticonvulsants, antidepressants, calcium channel blockers, and beta-blockers, but may not be fully effective. Anti-calcitonin gene-related peptide monoclonal antibody (CGRP-mAb) has dramatically changed the preventive treatment for patients with migraine who do not respond to these conventional oral prophylactic medicines [[18], [19], [20], [21], [22]]. Currently, three CGRP-mAbs are approved and available in Japan: galcanezumab, fremanezumab, and erenumab. European headache federation states that there are no data indicating if the use of CGRP-mAbs may favor detoxification in patients with chronic migraine and MOH [23]. However, the efficacy of CGRP-mAbs for chronic migraine and MOH has been demonstrated in subgroup analyses [[24], [25], [26]]. Recently, some studies corroborated the potential usefulness of CGRP-mAb treatment for these patients [[27], [28], [29], [30]]. However, few real-world studies have examined in detail the clinical course of headache symptoms and acute medication use (AMU) after starting CGRP-mAb treatment for patients with migraine with MOH without abrupt drug discontinuation and no hospitalization [30].

The aim of the present study was to evaluate the real-world clinical results of CGRP-mAb treatment for migraine with MOH without abrupt drug discontinuation and no hospitalization.

## 2 Materials and methods

### 2.1 Study design

This was a single-center, retrospective, real-world study of patients with migraine with MOH who received CGRP-mAb treatment. The patients were recruited from the specialized headache outpatient clinic at Nagoya Garden Clinic from May 2022 to December 2023. All patients had a diagnosis of migraine and MOH according to the International Classification of Headache Disorders 3 (ICHD-3) criteria [3]. Patients first underwent magnetic resonance imaging to exclude intracranial diseases, and then medical treatment for migraine was started. Diagnosis and treatment were performed by a neurosurgeon specializing in the field of pain and headaches (T.T.). CGRP-mAb treatment was started when the frequency of headaches or migraines did not decrease to the patient's satisfaction even after receiving one or more oral prophylactic medications, and the patients agreed to the treatment. All CGRP-mAbs were administered monthly, so no patients received fremanezumab quarterly dosing. Oral prophylactic medicines were given for at least 2 months prior to inclusion. Eligible patients for the outcome analysis met the following conditions: migraine with MOH continued even after receiving oral prophylactic treatment, naïve to CGRP-mAb treatment, and received initial CGRP-mAb injection by November 2023. This study was approved by the Ethics Review Committee of Nagoya University Graduate School of Medicine (approval number 2022-0316). Since this study was noninvasive, the Ethics Review Committee of Nagoya University Graduate School of Medicine approved that the requirement for written informed consent from patients was waived, but the opt-out method was adopted in accordance with the Japanese ethics guidelines. This research was completed in accordance with the Declaration of Helsinki as revised in 2013.

### 2.2 Data collection

Demographic data (age, sex, onset years of migraine, family history of headache, history of mental disturbance, migraine with aura, oral prophylactic medicines, and type of initial CGRP-mAb injection) were collected retrospectively. All patients recorded headache, migraine, and number of acute medication intakes in their headache diaries. A migraine day was defined as fulfilling the ICHD-3 criteria [3]. The following headache characteristics were compiled from the diary or electronic medical chart: monthly headache days (MHD), monthly migraine days (MMD), monthly AMU days, and monthly amount of AMU tablets. MMD counts only the number of migraine days per month, whereas MHD counts all monthly headache days, including both migraine and non-migraine headache days. Triptan and non-triptan medications (e.g. non-steroidal anti-inflammatory drugs, acetaminophen, and over-the-counter drugs) were considered acute medication, and the monthly amount of each and total AMU tablets were calculated. The choice of conventional oral prophylactic medicines was based on the experience of the treating physician. When patients agreed to start one of the three types of CGRP-mAb treatment, the choice of medication was determined through discussions between the patient and physician in the clinical setting. Actual use of acute medications was left to the patient without forcing abrupt drug discontinuation. The physician continued to provide information that it would be best to refrain from using acute medications if possible to patients before starting and during CGRP-mAb treatment. CGRP-mAb treatment was performed without hospitalization.

Data for MHD, MMD, AMU days, and amounts of AMU tablets were collected before starting CGRP-mAb treatment (baseline) and 1 month after the first (1st), second (2nd), and third (3rd) CGRP-mAb injections. The questionnaires of the headache impact test-6 (HIT-6), a migraine-specific quality of life questionnaire (MSQ) were also completed at the same time as above. Additionally, participants were asked to a simple questionnaire assessing their anxiety about headache attacks using a score of 1 or 0; 1 indicating having anxiety about headache attacks and 0 indicating no anxiety. This simple questionnaire did not ask about the daily problems caused by migraines.

### 2.3 Assessments and statistical analysis

The primary endpoint of this study was to determine whether there was a reduction of MHD after the 3rd CGRP-mAb injection compared to baseline in the target patients. The Shapiro-Wilk test was used to assess normality. Subsequently, the Wilcoxon signed-rank sum test was applied. In addition, a general linear mixed-effects model was used to examine whether MHD decreased at 1, 2, and 3 months post-CGRP-mAb use compared to baseline. This model included random effects for individuals and fixed effects for time intervals in an unstructured model. Similar analyses were conducted for secondary assessments, which included evaluating MMD, AMU days, total amount of AMU tablets, HIT-6 score, and MSQ score. In addition, a general linear mixed-effects model was used to examine whether MHD decreased at 1, 2, and 3 months post-CGRP-mAb use compared to baseline. The post-hoc test for pairwise comparisons between time points was conducted using Tukey's Honest Significant Difference Test. This model included random effects for individuals and fixed effects for time intervals in an unstructured model. Regarding sample size determination, it was challenging to estimate the extent to which CGRP-mAb treatment without abrupt drug discontinuation and no hospitalization reduced headache days for patients with MOH based on previous publications. Thus, this study retrospectively collected consecutive patients for analysis. Significance was set at p < 0.05. The statistical analyses were carried out using R version 4.1.2 (R Foundation for Statistical Computing, Vienna, Austria) and RStudio (version 2022.02.0; RStudio, Inc., 2022 Boston, MA, USA).

MHD and MMD reduction rates comparing baseline and after each CGRP-mAb injection were calculated. The reduction rates were classified into the following 3 categories: ≥75 %, <75 % and ≥50 %, and <50 %. A good responder to CGRP-mAb treatment was defined as a reduction rate ≥50 %. Reduction rates of monthly AMU days and total amount of AMU tablets were also compared from baseline to after each CGRP-mAb injection. The reduction rates were classified as follows: ≥75 %, <75 % and ≥50 %, and <50 %. Then, the results for AMU were defined as the following 3 categories: excellent, both items ≥75 %; good, both items <75 % and ≥50 %/one item <75 % and ≥50 % and the other item ≥75 %; and poor, either item was <50 %. Excellent and good were defined as “effective in reducing AMU”. The HIT-6 score and MSQ score after each CGRP-mAb injection were compared with baseline. The percentages of anxiety about headache symptoms before and after each CGRP-mAb injection were calculated. The number of patients who requested discontinuation during the observation period after starting CGRP-mAb treatment and the reasons for it were investigated.

## 3 Results

### 3.1 Participants’ demographic characteristics and baseline parameters

**Fig. 1:**
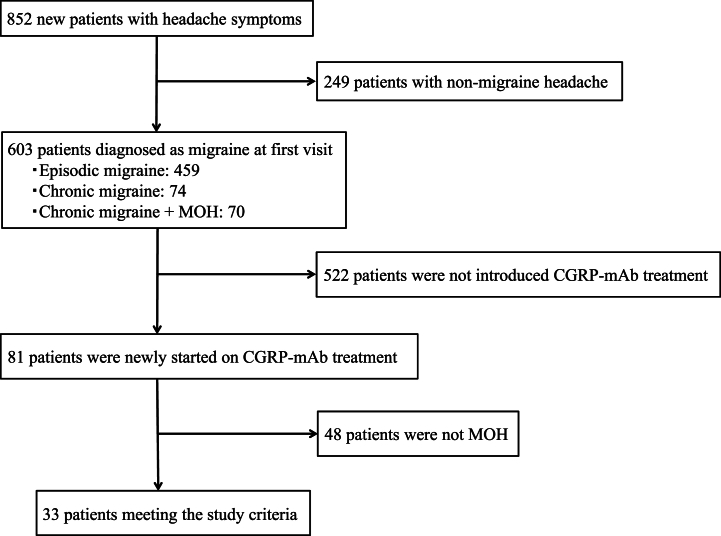
Inclusion criteria. CGRP-mAb: anti-calcitonin gene-related peptide monoclonal antibody, MOH: medication overuse headache.

From May 2022 to December 2023, 852 new patients with headache symptoms visited the specialized headache outpatient clinic, and 603 of them (70.8 %) were diagnosed as having migraine. The numbers of migraine types were as follows: episodic migraine 459; chronic migraine 74; and chronic migraine with MOH 70. Of the 603 patients with migraine, 81 (13.4 %) were newly started on CGRP-mAb treatment. There were 33 patients with chronic migraine with MOH who met the study criteria (Fig. 1). The clinical characteristics of the patients are shown in Table 1. The mean age was 40.9 ± 11.4 years, and the female-to-male ratio was 29:4. The onset ages of migraine were teens and younger (n = 23, 70.0 %), 20s (n = 3, 9.1 %), 30s (n = 6, 18.1 %), and 40s and older (n = 1, 3.0 %). Twenty-five patients had a family history of headaches (75.8 %), and 6 patients had a history of psychiatric disorders (18.2 %): depression in 4 (12.1 %) and anxiety in 2 (6.1 %). The type of migraine was migraine with aura in 17 patients (51.5 %). The mean number of types of oral prophylactic medicines used before starting CGRP-mAb treatment was 1.7, including anticonvulsants (27), antidepressants (14), calcium channel blockers (11), and beta-blockers (3). The CGRP-mAbs selected were galcanezumab 14, fremanezumab 13, and erenumab 6 (Table 1). No patients experienced side effects related to CGRP-mAb treatment, such as injection site reactions or constipation, and no patients dropped out due to side effects.

**Table 1:** Demographic and clinical characteristics of the patients.

Characteristics	n = 33
Age (years), mean ± SD	40.9 ± 11.4
Sex, female; n (%)	29 (87.9 %)
Onset years; n (%)	
Teens and younger	23 (70.0 %)
20s	3 (9.1 %)
30s	6 (18.1 %)
40s and older	1 (3.0 %)
Family history of headaches; n (%)	25 (75.8 %)
History of psychiatric disorders; n (%)	6 (18.2 %)
Depression	4 (12.1 %)
Anxiety	2 (6.1 %)
Migraine with aura; n (%)	17 (51.5 %)
Mean type of previous oral prophylactic drugs	1.7
Anticonvulsants	27 (81.8 %)
Antidepressants	14 (42.4 %)
Ca channel blockers	11 (33.3 %)
Beta blockers	3 (9.1 %)
Types of CGRP-mAb	
Galcanezumab	14 (42.4 %)
Fremanezumab	13 (39.4 %)
Erenumab	6 (18.2 %)

Ca: calcium, CGRP-mAb: anti-calcitonin gene-related peptide monoclonal antibody, n: number, SD: standard deviation.

### 3.2 Efficacy for MHD and MMD

Of the 33 patients enrolled, 27 continued CGRP-mAb injections 3 times. Six patients discontinued injections midway; 3 patients were lost during follow-up, 2 patients did not respond to CGRP-mAb and did not wish to continue the treatment, and 1 patient discontinued all prophylactic medications because she was planning to become pregnant. Data were missing for 3 patients who were lost to follow-up midway. One patient received three consecutive CGRP-mAb injections, but did not reach the assessment time of one month after the 3rd injection during the study period. Therefore, data on MHD, MMD, and AMU for this patient are available from baseline through the 2nd injection, but data after the 3rd injection is missing. Data on headache and acute medications were obtained from headache diaries in 30 patients (90.9 %), excluding 3 patients who were lost to follow-up midway.

**Fig. 2:**
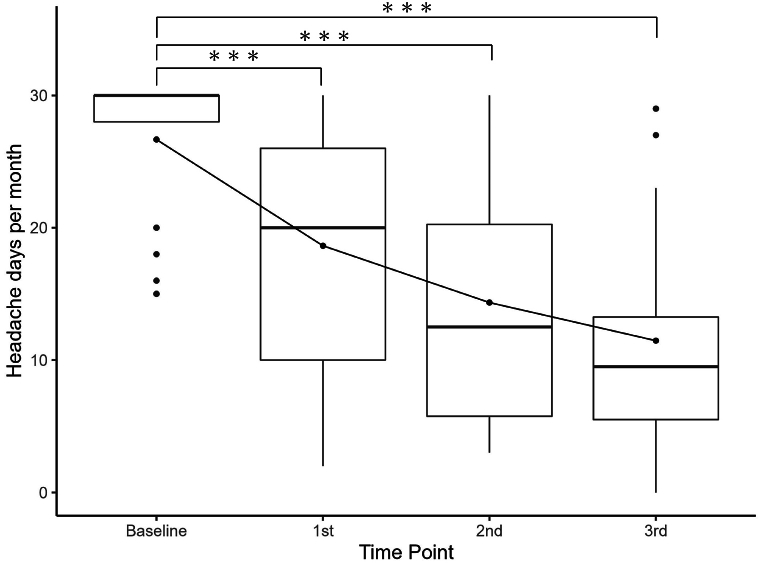
Changes in MHD before and after each CGRP-mAb injection. The boxes represent the 25 % and 75 % interquartile ranges, and the thick line inside the box represents the median. The mean values are expressed as a line graph. ∗∗∗: Significant differences compared with baseline, p < 0.001. CGRP-mAb: anti-calcitonin gene-related peptide monoclonal antibody, MHD: monthly headache days, 1st: first, 2nd: second, 3rd: third.

**Fig. 3:**
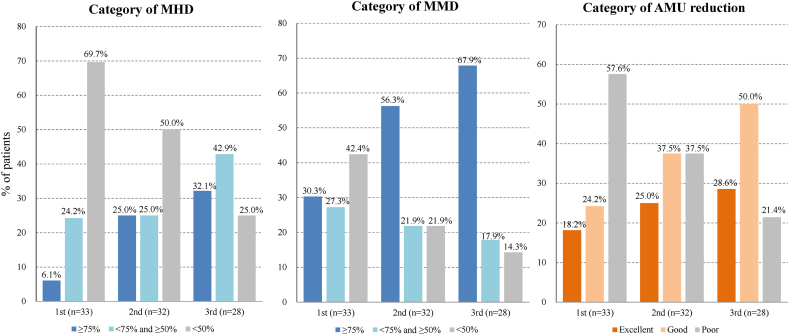
The percentages of patients according to the category of MHD, MMD, and AMU. The vertical axis represents the percentage of patients in each category. AMU: acute medication use, MHD: monthly headache days, MMD: monthly migraine days, n: number, 1st: first, 2nd: second, 3rd: third.

The MHD changes are shown in Fig. 2. A graph depicting the MHD changes using a general linear mixed-effects model and the results of the post-hoc analyses are shown in Supplementary Fig. 1 and Supplementary Table 1. The primary endpoint of MHD after the 3rd CGRP-mAb injection was median 9.5 [interquartile range (IQR), 5.5–13.3] days, which was significantly decreased from median 30.0 [IQR, 28.0–30.0] days at baseline. Results for MHD and MMD before and after each CGRP-mAb injection are shown in Table 2. MHD and MMD after each injection were significantly decreased from baseline (p < 0.001). The percentages of patients by category of MHD and MMD are shown in the bar graphs of Fig. 3. After the 3rd CGRP-mAb injection, number of patients of MHD categories were 9 (≥75 %), 12 (<75 % and ≥50 %), and 7 (<50 %), and that of MMD categories were 19 (≥75 %), 5 (<75 % and ≥50 %), and 4 (<50 %). Therefore, the good responder rates were 75.0 % (21/28) for MHD and 85.7 % (24/28) for MMD.

**Table 2:** Results of MHD, MMD, and AMU comparing before and after each CGRP-mAb injection.

	Before	After CGRP-mAb injection		
		1st (n = 33)	2nd (n = 32)	3rd (n = 28)
MHD	30.0 [28.0–30.0]	20.0∗∗∗ [10.0–26.0]	12.5∗∗∗ [5.8–20.3]	9.5∗∗∗ [5.5–13.3]
Good responder of MHD	–	10 (30.3 %)	16 (50.0 %)	21 (75.0 %)
MMD	10.0 [6.0–15.0]	3.0∗∗∗ [1.0–6.0]	1.5∗∗∗ [0.0–4.8]	1.5∗∗∗ [0.0–3.0]
Good responder of MMD	–	19 (57.6 %)	25 (78.1 %)	24 (85.7 %)
Total AMU days	28.0 [20.0–30.0]	15.0∗∗∗ [7.0–24.0]	9.5∗∗∗ [4.0–16.8]	8.0∗∗∗ [4.8–11.3]
Triptans	12.0 [7.5–20.0]	6.0 [4.0–9.0]	4.0 [3.0–8.5]	6.0 [2.3–8.0]
Non-triptans	25.0 [17.5–30.0]	16.0 [4.0–23.3]	7.0 [3.5–17.5]	5.0 [1.5–7.0]
Total amount of AMU tablets	30.0 [22.0–54.0]	17.0∗∗∗ [7.0–31.0]	11.0∗∗∗ [4.0–17.5]	9.5∗∗∗ [4.8–13.5]
Triptans	14.0 [7.5–20.5]	6.0 [3.5–9.5]	4.0 [3.0–9.0]	6.0 [2.3–8.8]
Non-triptans	30.0 [19.5–60.0]	15.5 [4.0–28.5]	8.0 [3.5–19.0]	5.0 [1.5–7.0]
Effective rates of AMU reduction	–	14 (42.4 %)	20 (62.5 %)	22 (78.6 %)

Data are expressed as median [interquartile ranges] or number (%), AMU: acute medication use, CGRP-mAb: anti-calcitonin gene-related peptide monoclonal antibody, MHD: monthly headache days, MMD: monthly migraine days, n: number, 1st: first, 2nd: second, 3rd: third, ∗∗∗: p < 0.001.

### 3.3 Efficacy for AMU

**Fig. 4:**
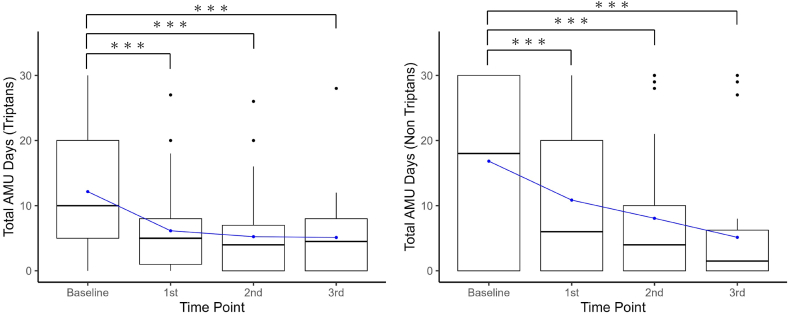
Changes of AMU days of each triptans and non-triptans. The boxes represent the 25 % and 75 % interquartile ranges, and the thick line inside the box represents the median. The mean values are expressed as line graphs. ∗∗∗: Significant differences compared with baseline, p < 0.001. AMU: acute medication use, 1st: first, 2nd: second, 3rd: third.

Results for AMU before and after each CGRP-mAb injection are also shown in Table 2. AMU days and tablets after each injection were significantly decreased from baseline (p < 0.001). After the 3rd CGRP-mAb injection, AMU days were significantly decreased from median 28.0 [IQR, 20.0–30.0] days to 8.0 [IQR, 4.8–11.3] days. In addition, the total amount of AMU was also significantly decreased, from median 30.0 [IQR, 22.0–54.0] tablets to 9.5 [IQR, 4.8–13.5] tablets. After the 3rd injection, the reductions of AMU days were 8 (≥75 %), 14 (<75 % and ≥50 %), 6 (<50 %), and those of the total amount of AMU tablets were 9 (≥75 %), 15 (<75 % and ≥50 %), and 4 (<50 %), respectively. The percentages of patients by category of AMU are shown in the bar graphs of Fig. 3. After the 3rd CGRP-mAb injection, the number of patients of AMU category was excellent in 8, good in 14, and poor in 6. Therefore, the rate of reducing AMU was 78.6 % (22/28). The changes of AMU days and the amount of AMU tablets each triptans and non-triptans drugs are shown in Fig. 4 and Supplementary Fig. 2, respectively. Patients who responded ≥75 % to the 1st CGRP mAb injection had a significantly reduction in acute medications, but none were able to discontinue them completely.

### 3.4 Changes of HIT-6 and MSQ and the presence of anxiety about headache symptoms

HIT-6 scores before and after the 1st, 2nd, and 3rd CGRP-mAb injections were median 63.0 [IQR, 61.0–66.0], 56.0 [IQR, 52.0–61.0], 53.0 [IQR, 48.0–58.8], and 56.0 [IQR, 48.0–59.5], respectively. MSQ scores before and after the 1st, 2nd, and 3rd injections were median 35.0 [IQR, 28.0–41.0], 21.0 [IQR, 19.0–28.0], 21.0 [IQR, 18.0–24.0], and 22.0 [IQR, 17.5–25.0], respectively. HIT-6 and MSQ scores were significantly decreased from baseline after each CGRP-mAb injection (p < 0.001). The percentage of anxiety about headache symptoms before, after 1st, 2nd, and 3rd CGRP-mAbs injection were 87.9 %, 69.7 %, 53.1 %, and 46.4 %, respectively.

## 4 Discussion

The present study showed the real-world clinical results of CGRP-mAb treatment for migraine with MOH without abrupt drug discontinuation and no hospitalization. After the 3rd CGRP-mAb injection, the primary endpoint of MHD was significantly decreased from median 30.0 [IQR, 28.0–30.0] days to 9.5 [IQR, 5.5–13.3] days. Secondary endpoints of MMD, AMU days, total amount of AMU tablets, HIT-6 score, and MSQ score were also significantly decreased compared to baseline. Good MHD and MMD responses were obtained in 75.0 % and 85.7 %, respectively. The rate of reducing AMU was 78.6 %.

There are still few real-world studies of CGRP-mAb treatment for migraine with MOH, and its effectiveness remains unclear [[27], [28], [29], [30]]. In a study investigating the effectiveness of CGRP-mAb treatment for MOH patients who were not using conventional oral prophylactic medicines or using low doses, 242 (80 %) of the 303 patients showed both a ≥50 % reduction of MHD and ≥50 % reduction of AMU days 3 months after CGRP-mAb treatment [28]. Pensato et al. compared one group with hospitalization and abrupt discontinuation of overused acute medication and another group with no hospitalization and no abrupt drug discontinuation, as a condition before starting CGRP-mAb treatment [30]. The latter group did not undergo abrupt drug discontinuation while hospitalized because of personal reasons or limitations by the COVID-19 pandemic. After 3 months of treatment, 51 % of the two groups achieved ≥50 % reduction in MHD, with no significant difference between the two groups. The result suggested that CGRP-mAb treatment may be effective for patients with MOH irrespective of abrupt drug discontinuation. Krymchantowski et al. reported comparing one group with treatment of CGRP-mAb added to abrupt drug discontinuation to another group with abrupt drug discontinuation alone for MOH [27]. After 3 months of treatment, both groups showed decreased MHD and monthly AMU days, although the former group had a greater reduction. Although abrupt discontinuation of overused medications with hospitalization is the gold standard treatment for MOH, it is more likely that patients can withdraw from MOH if CGRP-mAb treatment is started, regardless of abrupt drug discontinuation or hospitalization.

The present study evaluated in detail the clinical course of headache symptoms and acute medication use and found two interesting results. One result was the improvement of MHD and MMD. MHD decreased continuously from after the 1st to the 3rd CGRP-mAb injection, and the good responder rate finally exceeded 70 % after the 3rd injection. However, MMD were significantly decreased after the 1st injection, and the good responder rates were maintained over 70 % after the 2nd injection. The other result was the reduction of triptans and non-triptans. The number of days and amount of non-triptans use decreased continuously from after the 1st to the 3rd CGRP-mAbs injections. However, those of triptans decreased by approximately half after the 1st injection, although the decrease was less after the 2nd and 3rd injections. Drastic MMD and MHD reductions after the 1st CGRP-mAb injection led to significant reductions of both triptans and non-triptans. The reductions of migraine attacks and headaches reduced patients’ anxiety about headache symptoms, which gave them the courage to voluntarily refrain from taking non-triptans, which were customarily abused.

The present study has serious limitations that require discussion. First, this was a single-center, retrospective, small case series, and it did not demonstrate the effectiveness of CGRP-mAb treatment compared to a control group. Second, the three CGRP-mAbs were not randomly assigned, but they were selected clinically by the physician and patients. Third, criteria were not set for the timing of starting CGRP-mAb treatment, although the treatment was started after at least 2 months of oral prophylactic medicines. Fourth, after starting CGRP-mAb treatment, patients themselves decided when and how much acute medications to take. Therefore, acute medication use may reflect the patient's personality and living environment. Fifth, since this study had a short follow-up period because the endpoint was set after the 3rd CGRP-mAb injection, long-term results are still unclear. Finally, anxiety about headache attacks was assessed using a simple questionnaire scored 0 or 1. The results of this questionnaire were used as a reference data but did not provide an accurate assessment of anxiety state.

## 5 Conclusions

The present study showed the real-world clinical results of CGRP-mAb treatment for migraine with MOH without abrupt drug discontinuation and no hospitalization. After the 3rd CGRP-mAb injection, MHD decreased significantly from median 30.0 [IQR, 28.0–30.0] days to 9.5 [IQR, 5.5–13.3] days. Good MHD and MMD responses were obtained in 75.0 % and 85.7 %, respectively, and the rate of reducing AMU was 78.6 %.

## CRediT authorship contribution statement

**Takafumi Tanei:** Writing – original draft, Resources, Project administration, Formal analysis, Data curation, Conceptualization. **Yutaro Fuse:** Formal analysis, Data curation, Conceptualization. **Satoshi Maesawa:** Writing – review & editing, Supervision, Methodology. **Yusuke Nishimura:** Writing – review & editing, Supervision. **Tomotaka Ishizaki:** Writing – review & editing, Supervision. **Yoshitaka Nagashima:** Writing – review & editing, Methodology. **Manabu Mutoh:** Methodology, Data curation. **Yoshiki Ito:** Methodology, Data curation. **Miki Hashida:** Methodology, Formal analysis, Data curation. **Takahiro Suzuki:** Methodology, Data curation. **Syun Yamamoto:** Methodology, Formal analysis, Data curation. **Toshihiko Wakabayashi:** Supervision, Project administration, Conceptualization. **Ryuta Saito:** Supervision, Resources, Project administration, Methodology, Investigation, Conceptualization.

## Ethics approval and consent to participate

This study was approved by the Ethics Review Committee of Nagoya University Graduate School of Medicine (approval number 2022-0316). Since this study was noninvasive, the Ethics Review Committee of Nagoya University Graduate School of Medicine approved that the requirement for written informed consent from patients was waived, but the opt-out method was adopted in accordance with the Japanese ethics guidelines.

## Consent for publication

All authors read and approved the final manuscript.

## Availability of data and materials

The datasets used and/or analyzed during the current study are available from the corresponding author on reasonable request.

## Funding

None.

## Declaration of competing interest

TT received speaking fees from Daiichi Sankyo, Otsuka Pharmaceutical, and Amgen. YF reports no conflict of interest. SM reports no conflict of interest. YN reports no conflict of interest. TI reports no conflict of interest. YN reports no conflict of interest. MM reports no conflict of interest. YI reports no conflict of interest. MH reports no conflict of interest. TS reports no conflict of interest. SY reports no conflict of interest. TW reports no conflict of interest. RS received speaking fees from Daiichi Sankyo.
