# Supplementary figures and images for: Real-world clinical results of CGRP monoclonal antibody treatment for medication overuse headache of migraine without abrupt drug discontinuation and no hospitalization
Source: Heliyon. 2024 Nov 6;10(22):e40190. doi: 10.1016/j.heliyon.2024.e40190 (PMC11693917; doi:10.1016/j.heliyon.2024.e40190)

## Slide 1
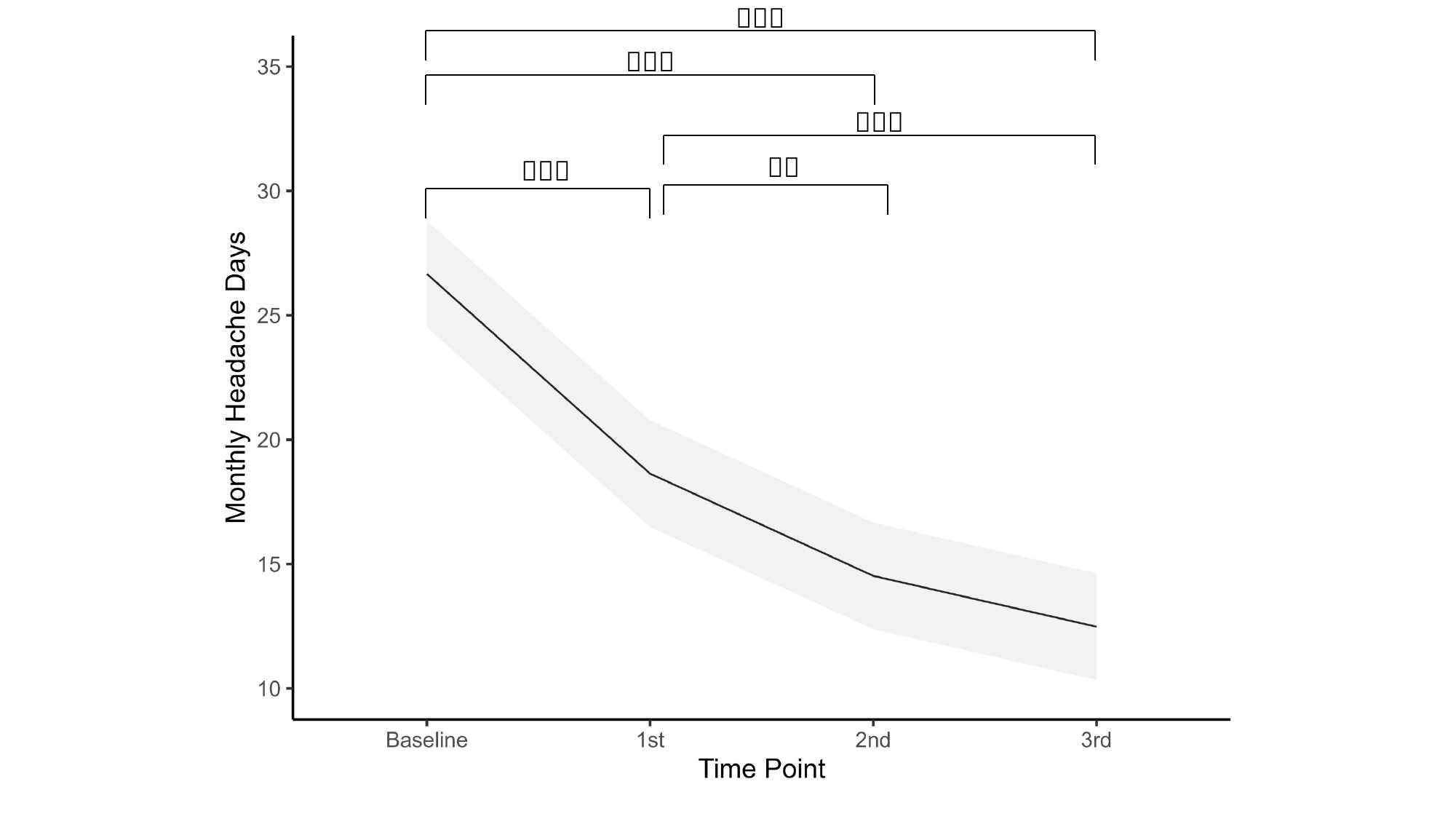

＊＊＊
＊＊＊
＊＊＊
＊＊
＊＊＊

Supplement: Multimedia component 1 [file mmc1.pptx]

## Slide 1
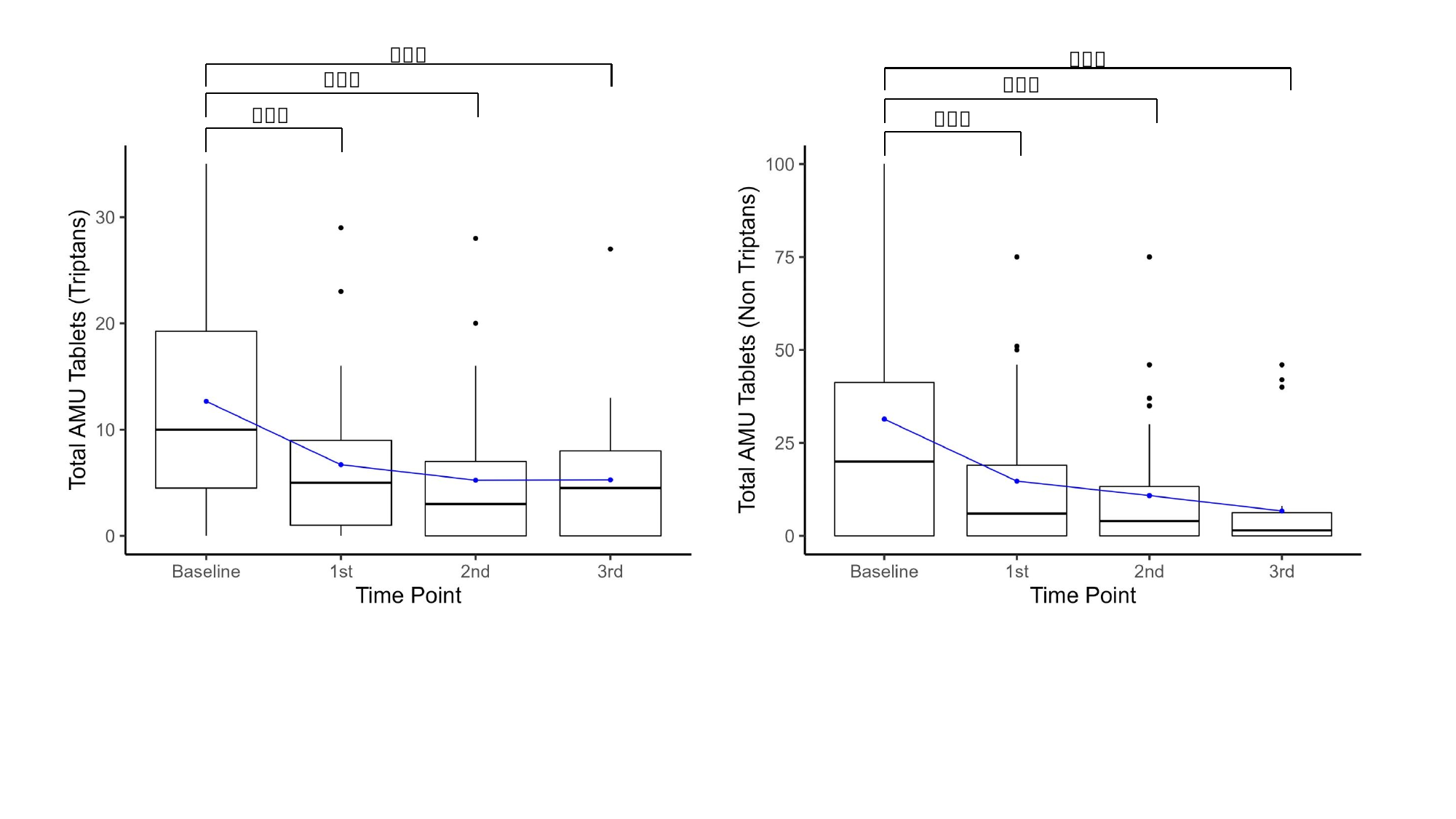

＊＊＊
＊＊＊
＊＊＊
＊＊＊
＊＊＊
＊＊＊

Supplement: Multimedia component 2 [file mmc2.pptx]
