# Supplementary material for: Real-world clinical results of CGRP monoclonal antibody treatment for medication overuse headache of migraine without abrupt drug discontinuation and no hospitalization
Source: Heliyon. 2024 Nov 6;10(22):e40190. doi: 10.1016/j.heliyon.2024.e40190 (PMC11693917; doi:10.1016/j.heliyon.2024.e40190)
Supplement: Multimedia component 3 [file mmc3.docx]

| Supplementary Table 1. Pairwise Comparisons of Time Points with Tukey’s HSD Adjustment | | | | | |
| --- | --- | --- | --- | --- | --- |
| Contrast | Estimate | Standard Error (SE) | Degrees of Freedom (df, Kenward-Roger) | t-ratio | p-value |
| Baseline - 1st | 8.03 | 1.19 | 90 | 6.77 | **<.0001** |
| Baseline - 2nd | 12.14 | 1.2 | 90.3 | 10.124 | **<.0001** |
| Baseline - 3rd | 14.18 | 1.25 | 90.8 | 11.314 | **<.0001** |
| 1st - 2nd | 4.11 | 1.2 | 90.3 | 3.428 | **0.0050** |
| 1st - 3rd | 6.15 | 1.25 | 90.8 | 4.909 | **<.0001** |
| 2nd - 3rd | 2.04 | 1.26 | 90.6 | 1.624 | 0.3706 |
